# Editorial: New challenges with the management of rheumatic heart disease

**DOI:** 10.3389/fsurg.2022.1030172

**Published:** 2022-10-11

**Authors:** Amiliana M. Soesanto

**Affiliations:** Department Cardiology and Vascular Medicine, Faculty of Medicine, Universitas Indonesia, National Cardiovascular Center Harapan Kita, Jakarta, Indonesia

**Keywords:** group a streptococcus, rheumatic heart disease, screening, ACE inhibitor, secondary prophylaxis

**Editorial on the Research Topic** New challenges with the management of rheumatic heart disease By Soesanto AM. (2022) Front. Surg. 9: 1030172. doi: 10.3389/fsurg.2022.1030172

Rheumatic Heart Disease (RHD) is still a significant health problem, especially in low- and middle-income countries, with 40.5 million prevalent cases resulting in ≈305,000 deaths annually (1, 2). This editorial has covered the articles from the low-middle income countries related to the problem of RHD through the original research and reviews. Unfortunately, only four of the seven articles submitted to the Research Topic are relevant to RHD, preventing a more in-depth understanding of the subject. The low submission rate could be attributed to the difficulties that low- and middle-income countries face in conducting qualified studies. The majority of large RHD studies are multicenter and led by experts from developed countries with adequate funding. A financial constraint limits the methodology and size of the study in low/middle-income countries, not to mention the budget for publication. A language barrier could also make writing and publishing a scientific manuscript difficult. Collaboration with international organizations and between countries may be a good way to increase the number of RHD studies in endemic countries. Follows are the editorial of the interesting four articles related with RHD.

Group A Streptococcus (GAS) infection may cause acute rheumatic fever (ARF) and further lead to a devastating complication, rheumatic heart disease (RHD) (3). The World Health Organization (WHO) recommends that all patients with confirmed RHD receive secondary prophylaxis against repeated attacks of ARF in order to prevent further valvular damage (4). The paper by Sarah Wangilisasi et al. (2020) reported a low proportion of RHD patients on regular secondary prophylaxis. Further, GAS throat colonization is high among this population and is associated with not taking/stopping the prophylaxis regimen. Similar founding also reported in a systematic review and meta-analysis that GAS infection is still a global burden (5). This study also reported that most (96%) of GAS isolates were susceptible to penicillin. Based on their findings, educating patients and physicians to use effective secondary prophylaxis is crucial. Another large trial, the GOAL trial, showed that secondary prophylaxis using penicillin reduced the risk of the progression of latent rheumatic heart disease in children and adolescents (6). Improving delivery of secondary prophylaxis can be achieved by strategies to support culturally and age-appropriate adherence and more patient-centered approaches within culturally competent health systems (7).

Another study from a tertiary cardiac center reported the profile and management of RHD. Oktavia Lilyasari et al. (2020) found that RHD complication is most commonly pulmonary hypertension and atrial fibrillation in young adults and heart failure in children. Reactivation cases were found in 17% of cases. Multiple valve disease was more frequent than single valve disease, which may create a heavier hemodynamic burden on the heart. This study showed that even children and young adults already experienced serious complications of RHD and need cardiac surgery-most commonly valve repair. As the disease progresses, a second or even more re-operation may be needed with more challenging surgical techniques, possibly increasing surgical risk. The patients may have a risk of thromboembolism and need life-long anticoagulation, which further exposes them to the risk of bleeding. A high-risk pregnancy also becomes one of the problems. RHD and its complications will cause long-life morbidity and poor outcome. Since this disease is preventable, again prophylaxis strategy is essential. For severe RHD or patients who have had cardiac surgery, although, in individuals >40 years old, the risk of recurrence is extremely low, some guidelines recommend continuing secondary/tertiary prophylaxis. It may be continued beyond 40 years old or even lifelong, especially for patients who have had cardiac valve surgery (8).

The earlier two articles above implied the importance of finding earlier stages of RHD to prevent any progression which leads to further disease burden and poor outcomes. A perspective by Amiliana M Soesanto et al. (2020) proposed a strategy to screen for latent RHD in Indonesia as an early step to control the disease progression. Applying simplified echo criteria, using handheld echo devices, training extended echo operators, and targeting specific groups of children are important variables for screening strategy. Perhaps this strategy may be applied in other low-middle income countries with similar limitations and problems. Nevertheless, to control RHD, it needs good collaboration among the community, health experts, and the government. A model for strengthening health systems is proposed to address other cardiovascular diseases in limited-resource countries. It may require a combination of the prevention of rheumatic fever and RHD, typically through primary healthcare services in community settings; advanced care, which includes tertiary cardiology and cardiac surgery services; and health policy, including measures that national health systems should take (9).

The progression of valve deterioration in RHD includes inflammation and fibrosis. However, the true pathologic mechanism has not been well understood. In the mini-review, Ade M Ambari et al. (2020) explained some hypotheses about the pathomechanism of valve fibrosis which involves angiotensin II, and the proposed mechanism of how angiotensin-converting enzyme (ACE) inhibitor may decrease the progression of valve fibrosis. He stated that TGF-b induction by Angiotensin II could further increase the binding of IL-33 to sST2, resulting in the upregulation of Angiotensin II and may progress to calcification and stiffening of the heart valves. ACEIs directly reduce the binding of IL-33 to sST2 and through the inhibition of TGF-b/MAPK/Smad signaling. Therefore, ACE inhibitors may potentially attenuate cardiac fibrosis in RHD *via* the IL-33/ST2 axis. Although ACE inhibitor is considered hemodynamically safe and effective in patients with rheumatic MR (10), recent valvular guidelines have not yet recommended the use of ACE inhibitor in the medical management of MS (11, 12). This interesting theory needs to be studied further to prove the protective effect on the progression of valve deterioration in RHD, and the authors have been doing an on-going randomized controlled trial to prove this theory (13). If positive results are consistently found, this may become a breakthrough and change the medical management strategy in rheumatic MS patients.

In summary, all four articles shared facts and information about the burden of RHD. It includes the importance of GAS infection, the advanced clinical features of RHD in the young, and the proposed theory causing progressive deterioration of the valves. These articles imply the need for prophylaxis action, starting from a screening program for latent RHD (Table 1). The screening program and prophylaxis action are quite challenging in low-middle income countries. However, a good collaboration among the community, health experts, and the government is important to control RHD better.

**Table 1:** Summary of the research topic “New Challenges with the Management of Rheumatic Heart Disease.”

Article title	Authors/DOI	Key points	Possible implication
Throat Colonization and Antibiotic Susceptibility of Group a b-Hemolytic Streptococci Among Rheumatic Heart Disease Patients Attending a Cardiac Referral Hospital in Tanzania, a Descriptive Cross-sectional Study	Sarah Wangilisasi et al. (2020) doi: 10.3389/fsurg.2020.00057	• The proportion of patients on regular secondary prophylaxis is unacceptably low • GAS throat colonization is high among this population and is associated with stopping prophylaxis • The majority (96%) of GAS isolates were susceptible to penicillin	• Need strategies to improve the delivery of secondary prevention. • Interventions should target both patients’ and physicians’
Clinical Profile and Management of Rheumatic Heart Disease in Children and Young Adults at a Tertiary Cardiac Center in Indonesia	Oktavia Lilyasari, et al. (2020) doi: 10.3389/fsurg.2020.00047	• Common complications include pulmonary hypertension, atrial fibrillation in young adults, and heart failure in children. • Reactivation cases were quite frequent. • Multiple valve lesions were more common than the single lesion. • Valve repair was preferable in children.	• Early detection is needed. • Secondary/tertiary prophylaxis is crucial with adequate duration
Echocardiography Screening for Latent Rheumatic Heart Disease: What Can We Do in Indonesia?	Amiliana M. Soesanto et al. (2020) doi: 10.3389/fsurg.2020.00047	• No prevalence data on RHD in Indonesia • Echocardiography screening is important • Simplified echo criteria, handheld echo device, an extension of trained echo operator, and screening target are important variables for screening strategy.	• A good and feasible strategy is needed to screen for latent RHD • Good collaboration among the community, health experts, and the government
Angiotensin Converting Enzyme Inhibitors (ACEIs) Decrease the Progression of Cardiac Fibrosis in Rheumatic Heart Disease Through the Inhibition of IL-33/sST2	Ade M Ambari et al. (2020) doi: 10.3389/fcvm.2020.00115	• Angiotensin II involves a cascade of inflammation, and valvular fibrosis causes calcification and stiffening of the heart valves in RHD. • ACE inhibitor results in cardioprotection against cardiac fibrosis in the pathogenesis of RHD *via* the IL-33/ST2 axis	• Further studies need to be done to prove this theory • If positive results are consistently found, this may change the medical therapy strategy in Rheumatic MS patients.
